# Surveillance of invasive pneumococcal disease in Germany: surge in PCV13 serotypes and new challenges, 2017 to 2024

**DOI:** 10.2807/1560-7917.ES.2026.31.21.2500576

**Published:** 2026-05-28

**Authors:** Mark PG van der Linden, Mathias W Pletz, Andreas Itzek

**Affiliations:** 1 Reference Laboratory for Streptococci, Department of Medical Microbiology, University Hospital Aachen (RWTH), Aachen, Germany; 2 Institute of Infectious Diseases and Infection Control and Center for Sepsis Care and Control, Jena University Hospital, Friedrich-Schiller-University Jena, Jena, Germany

**Keywords:** *Streptococcus pneumoniae*, invasive pneumococcal disease, pneumococcal conjugate vaccines, children, Germany

## Abstract

**BACKGROUND:**

Pneumococcal vaccination has been recommended in Germany for adults aged ≥ 60 years (1998) and children aged < 2 years (2006), resulting in a reduction of pneumococcal disease incidence in vaccinated children and unvaccinated individuals through herd protection.

**AIM:**

We aimed to investigate serotype distribution of invasive pneumococcal disease (IPD) among children and adults in 2017–2024.

**METHODS:**

We analysed data of an ongoing nationwide surveillance of IPD. All isolates were serotyped by Neufeld’s Quellung reaction.

**RESULTS:**

In total, 24,122 IPD isolates were analysed by the German Reference Laboratory for Streptococci. Over the study period, case numbers were lower during the COVID-19 pandemic and increased afterwards with higher proportion of pneumococcal conjugate vaccine (PCV)13 serotypes. Serotypes 22F and 33F, included in PCV15 and PCV20, showed only minor variations, whereas PCV20 unique serotypes were less frequently observed in 2022–2024. Serotype 3 persisted in IPD across all age groups, while serotype 4 disappeared in children and adolescents, but increased in adults, most prominently in 18–49-year-olds. Serotype 38 steeply increased in 2023/24, among children aged < 2 years and older adults.

**CONCLUSION:**

The concerning increase of PCV13 serotype IPD in Germany might be related to low vaccination uptake, resulting in inefficient herd protection. The dominance of serotype 3 and the rise of serotype 4 among adults highlight the importance of direct protection and support the current national vaccination recommendation for adults aged ≥ 60 years. The increase in serotype 38 IPD among young children and older adults might indicate a replacement phenomenon, highlighting the importance of IPD serotype surveillance.

**Key public health message**

**What did you want to address in this study and why?**

Pneumococci are bacteria that can cause ear, lung and brain infections. Vaccines protect against certain number of different serotypes of this bacterium and are recommended for those most at risk: young children and older adults. We are studying how well the current vaccination strategy prevents pneumococcal infections in Germany.

**What have we learnt from this study?**

We saw that some of the bacterial types included in vaccines used in Germany such as serotypes 3, 4, 8 and 22F are becoming more common. Also, some serotypes have increased, such as 24F and 38 that are not covered in any of the currently available vaccines.

**What are the implications of your findings for public health?**

The rise in serotypes that vaccines can prevent might be caused by not enough people getting vaccinated in Germany. The increase in vaccine preventable infections among adults shows the importance of direct vaccination protection and supports the current vaccination recommendation for adults 60 years and older. The appearance of new serotypes emphasises the importance of continuous monitoring.

## Introduction

Childhood conjugate vaccination has had a strong impact on pneumococcal disease incidence [1]. Nevertheless, *Streptococcus pneumoniae* is still a leading cause of lower respiratory tract infections [2] and invasive pneumococcal disease (IPD) [3,4]. The polysaccharide capsule of *S. pneumoniae* is described as the main virulence factor [5] with currently 107 different capsular types [6]. The antigenic spectrum of these types is addressed by different vaccine formulations (Table 1).

**Table 1:** Serotype composition of pneumococcal vaccines

Vaccine	Serotypes included
Pneumococcal conjugate vaccines (PCV)	
PCV7	4, 6B, 9V, 14, 18C, 19F, 23F
PCV10	PCV7 and serotypes 1, 5, 7F
PCV13	PCV10 and serotypes 3, 6A, 19A
PCV15	PCV13 and serotypes 22F, 33F
PCV20	PCV15 and serotypes 8, 10A, 11A, 12F, 15B
PCV24	PCV20 and serotypes 2, 9N, 17F, 20
PCV21 (newly licensed)	3, 6A, 7F, 8, 9N, 10A, 11A, 12F, 15A, 15C, 16F, 17F, 19A, 20, 22F, 23A, 23B, 24F, 31, 33F, 35B
Pneumococcal polysaccharide vaccine (PPV)	
PPV23	1, 2, 3, 4, 5, 6B, 7F, 8, 9N, 9V, 10A, 11A, 12F, 14, 15B, 17F, 18C, 19A, 19F, 20, 22F, 23F, 33F

The newly licensed PCV21 vaccine, intended for adults aged ≥ 18 years, has a unique serotype composition. It does not include any PCV7 serotypes nor serotypes 1, 5 and 15B but instead has 10 serotypes of PCV20, three serotypes of PPV23 and eight serotypes not included in any other licensed vaccine. Two new vaccines PCV24^MAPS^ and PCV24^eCRM^ are currently in development. The vaccines are presented in more detail in Supplementary Table S1.

In 2017–2024, PCV13 and, since May 2023, PCV15 have been recommended in Germany for all children aged < 2 years in a 2 + 1 schedule (two primary vaccine doses at the age of 2 and 4 months, followed by a booster dose at the age of 11 months) [7-9]. For adults aged ≥ 60 years, a general recommendation for vaccination with PPV23 has been in place since 1998 [10], which was changed to PCV20 in September 2023.

We have previously described the direct and indirect effects (herd protection) of PCV7, PCV10 and PCV13 childhood vaccination up to the pneumococcal season 2017/18 [11,12]. Unfortunately, the uptake of PCVs among children remains insufficient in Germany. In the seasons following the general vaccine recommendations of 2006, the uptake reached as high as 85% [13], but only 74% of children born in 2021 were fully vaccinated at the age of 2 years [14]. Among adults, PPV23 uptake has remained low ever since its introduction, at levels between 20 and 30% [14-16]. Here we describe the serotype dynamics of IPD among children and adults in Germany covering the pneumococcal seasons 2017/18 to 2023/24, with special focus on the ongoing national PCV-based vaccination strategy, the potential effectiveness of current and future vaccine formulations, as well as COVID-19 pandemic effects, caused by non-pharmaceutical interventions (NPIs) [17-19].

## Methods

### Study design

*Streptococcus pneumoniae* isolates from blood, cerebrospinal fluid or any other normally sterile body sites, isolated between 2017 and 2024 and submitted from 286 microbiological diagnostic laboratories located all over Germany, were included in the study, as described elsewhere [11]. Briefly, in Germany, microbiological diagnostic analyses are performed in both proprietary hospital laboratories and commercial laboratories serving the clinical departments of hospitals, with some large companies operating nationwide. The laboratory network continuously changes because of mergers, start-ups and hospitals changing their commercial providers. Around 90 new laboratories have joined the network since 2014. However, it is unclear how many of them involve mergers or new providers serving the same clinical departments, as this information is not publicly available. Between 2017/18 and 2021/22, 178, 184, 178, 141 and 174 laboratories sent samples to the German Reference Laboratory for Streptococci (GRLS), whereas in 2022/23 and 2023/24, 212 and 214 laboratories, respectively, submitted isolates to the GRLS. Following the introduction of the mandatory IPD notification, 43 additional laboratories joined the GRLS surveillance. However, these new laboratories submitted only 900 (8.2%) of the 11,000 isolates sent to the GRLS between 2022/23 and 2023/24.

Since 1997, diagnostic facilities have been requested to send isolates from patients with IPD to the GRLS with parallel reporting to the Robert Koch Institute (RKI). In March 2020, IPD became a notifiable condition in Germany, with mandatory laboratory reporting to local health authorities, while sample submission to the GRLS has not been changed.

### Isolate characterisation

Identification of *S. pneumoniae* was performed using bile solubility and optochin susceptibility testing, as well as sequence analysis of the genes *16S rRNA*, *cpsA*, *lytA,* *sodA* and *rpsB* [20-24]. Pneumococcal isolates were serotyped by Neufeld’s Quellung reaction (SSI Diagnostica Group, Hillerød, Denmark) [25]. This enabled identification of 97 of the currently known 107 serotypes, while 6H, 10D, 11E, 15D, 20A/B/C, 33E/G/H and 36A/B were not uniquely identifiable, and serogroups 20 and 36 were typed to the group level only. Isolates were considered non-typeable (NT) when there was no reaction with any of the antisera, but they were still reliably identified as *S. pneumoniae*.

### Data processing

Cases were grouped based on pneumococcal epidemiological season (from 1 July to 30 June of the following year) [26]. Seasonally reported IPD incidence rates of the GRLS surveillance were calculated using population data from the GENESIS-Online database (https://www-genesis.destatis.de/datenbank/online) of the German Federal Statistical Office (DESTATIS, https://www.destatis.de/EN/Home/_node.html), annually published on 31 December for the season, and compared with notification data of IPD from SurvStat@RKI 2.0 (https://survstat.rki.de/), for the same season.

The World Health Organization (WHO) declared COVID-19 as a pandemic on 11 March 2020 and declared the pandemic over on 5 May 2023 [27]. In this study, we categorised 2017/18, 2018/19 and 2019/20 as pre-pandemic seasons, 2020/21 as pandemic season, and 2021/22, 2022/23 and 2023/24 as post-pandemic seasons.

### Statistical analysis

Generalised linear models (GLM) with a binomial distribution and logit link, implemented in R (https://www.r-project.org; version 4.5.2; stats package), were used to assess whether the proportion of a serotype (or serotype group) among IPD isolates differed between pneumococcal seasons. Odds ratios (OR) with 95% confidence intervals (CI) were calculated to measure the association between season and the odds of an isolate belonging to a given serotype (or serotype group). An OR with a 95% CI excluding 1 was interpreted as evidence of a statistically significant difference in serotype distribution between specified seasons.

## Results

### Study cohort description

#### Reporting rates

**Figure 1:**
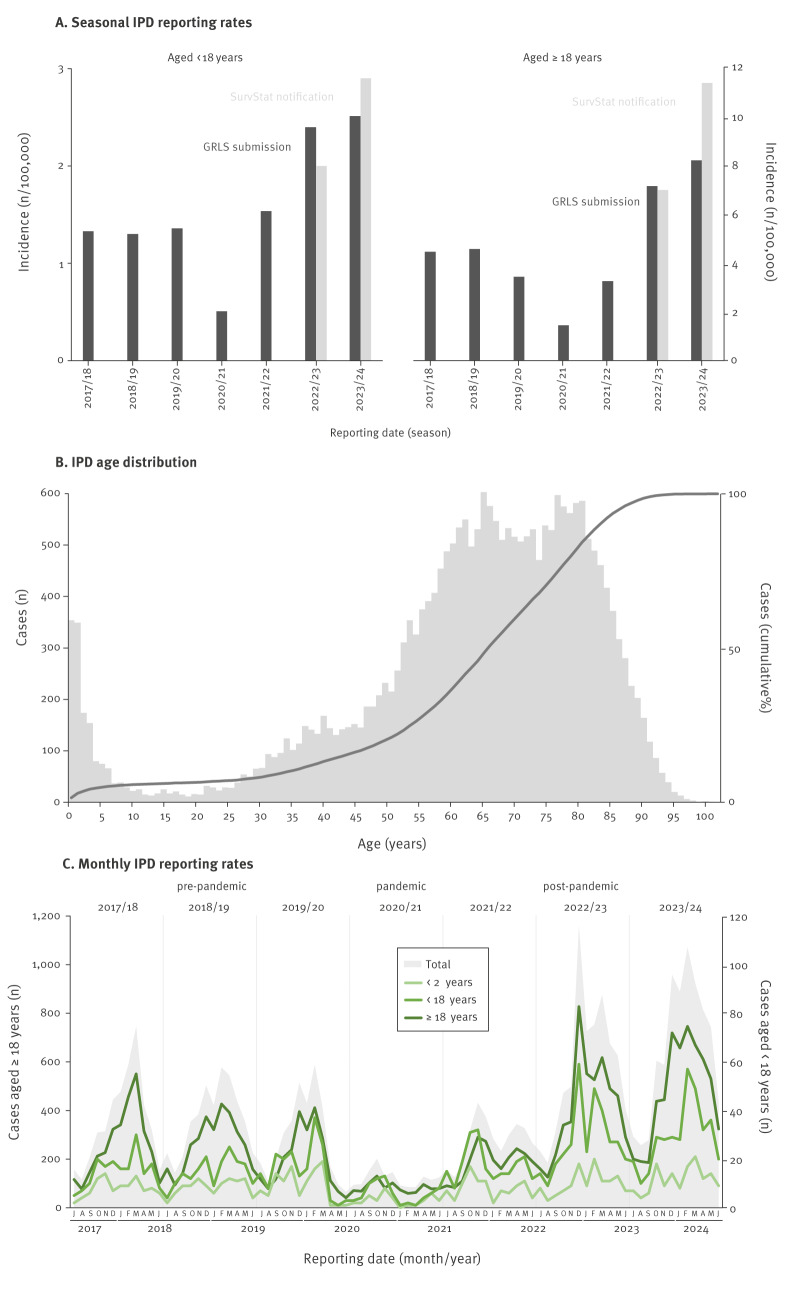
Reported numbers of cases with invasive pneumococcal disease (IPD), Germany, 2017/18–2023/24

GRLS: German Reference Laboratory for Streptococci; SurvStat: a web-based, public access database used to query aggregated surveillance data on notifiable infectious diseases in Germany.

Based on the GRLS data, the seasonally reported rates for IPD among children and adolescents aged < 18 years varied from 1.3 per 100,000 in 2017/18 to 0.5 per 100,000 in 2020/21 and increased to 2.4 per 100,000 in 2022/23 and to 2.5 per 100,000 in 2023/24 (Figure 1A). According to the SurvStat@RKI 2.0 database, laboratory notifications were 2.0 per 100,000 in 2022/23 and 2.9 per 100,000 in 2023/24, thus the capture rates of GRLS were 120% and 87%, respectively.

Panel A. Seasonal IPD incidence rates for children and adolescents aged < 18 years (left diagram) and adults aged ≥ 18 years (right diagram) derived from GRLS surveillance submission numbers (dark grey bars) and from SurvStat@RKI 2.0 laboratory notification numbers (light grey bars) by isolation date of the corresponding pneumococcal sample.

Panel B. IPD age distribution with total number of reported cases (light grey bars, left axis) and cumulative percentage (dark grey line, right axis).

Panel C. Monthly IPD reporting rates by age group with children aged < 2 years (light green line, right axis), children and adolescents aged < 18 years (medium green line, right axis), adults aged ≥ 18 years (dark green line, left axis), as well as total IPD case numbers (light grey area, left axis), with month and calendar year (below) and infection season and COVID-19 pandemic classification (above) based on the isolation date of the respective pneumococcal isolates.

In this study, we categorised 2017/18, 2018/19 and 2019/20 as pre-pandemic seasons, 2020/21 as pandemic season, and 2021/22, 2022/23 and 2023/24 as post-pandemic seasons.

For adults aged ≥ 18 years, seasonal IPD rates of the isolates submitted to GRLS were 4.5 per 100,000 in 2017/18, 1.4 per 100,000 in 2020/21, 7.2 per 100,000 in 2022/23 and 8.2 per 100,000 in 2023/24, with a capture rate of 102% and 72%, when compared with the corresponding SurvStat notifications of 7.0 per 100,000 in 2022/23 and 11.4 per 100,000 in 2023/24. In 2023/24, IPD rates of submitted isolates were highest in children aged < 2 years and adults aged ≥ 60 years (Table 2).

**Table 2:** Numbers of submitted isolates from patients with invasive pneumococcal disease (IPD) (n = 6,065) and reported cases of IPD (n = 8,292), by age, Germany, 2023/24

Age (years)	GRLS surveillance		SurvStat notification	
	Cases (n)	Cases (n/100,000)	Cases (n)	Cases (n/100,000)
< 2	139	9.7	141	9.9
2–4	105	4.4	124	5.2
5–17	107	1.1	140	1.4
18–49	742	2.3	1,062	3.3
50–59	691	5.6	961	7.8
60–75	2,141	12.9	2,901	17.4
> 75	2,140	25.5	2,963	35.2
Alternative grouping				
< 5	244	6.4	265	7.0
5–64	2,149	3.5	3,031	5.0
> 64	3,672	19.6	4,996	26.7
< 18	351	2.5	405	2.9
≥ 18	5,714	8.2	7,887	11.4

GRLS: German Reference Laboratory for Streptococci; SurvStat: electronic reporting system for surveillance of notifiable infectious diseases in Germany.

Over the study period, 26,441 isolates from patients with IPD were submitted to the GRLS. A total of 2,319 (8.8%) isolates were excluded from the study: 1,402 (5.3%) could not be cultured upon arrival, 440 (1.7%) were heavily contaminated, 351 (1.3%) were not identified as *S. pneumoniae*, 117 (0.4%) were not from German citizens and 9 (0.03%) did not have traceable data on patient age. For the season 2023/24, this resulted in 687 (10.2%; 687/6,746) excluded isolates. When taking these excluded isolates into account, capture rates for 2023/24 were 98% for children and adolescents and 80% for adults.

#### Patient characteristics

From 1 July 2017 to 30 June 2024, 24,122 isolates from patients with IPD were included in the study, comprising 1,511 (6.3%) from children and adolescents aged < 18 years (< 2 years: n = 703 (2.9%); 2–4 years: n = 408 (1.7%); 5–17 years: n = 400 (1.7%)) and 22,611 from adults aged ≥ 18 years (18–49 years: n = 2,912 (12.9%); 50–59 years: n = 3,075 (12.7%); 60–75 years: n = 8,397 (34.8%); > 75 years: n = 8,227 (34.1%)) (Figure 1B). Of the patients with available data on sex (n = 23,825; 98.8%), 13,342 (56.0%) were male (< 18 years: 55.4%; ≥ 18 years: 56.1%) and 10,483 (44.0%) were female.

#### Seasonal distribution

In the study period, IPD case numbers were always higher in winter months and decreased in 2020/21, during the COVID-19 pandemic, which started to spread in Germany in March 2020 (Figure 1C). Case numbers in children and adolescents returned to pre-pandemic levels in June 2021, for adults in August 2021, with a second drop between December 2021 and April 2022. In the post-pandemic seasons 2022/23 and 2023/24, a general increase in reported cases was observed, except for children aged < 2 years, as presented in Supplementary Table S2.

Among children and adolescents aged < 18 years, an average of 181 cases were reported per season in the pre-pandemic periods, which decreased to 70 cases during the COVID-19 pandemic and increased to a post-pandemic average of 299, as presented in Supplementary Table S3. Average pre-pandemic case numbers in adults aged ≥ 18 years were 2,890, which decreased to 1,002 cases in the pandemic season, followed by an initial increase in 2021/22 to 2,266 cases, and a marked rise in 2022/23 (n = 4,958) and 2023/24 (n = 5,714).

### Serotype dynamics

#### Vaccine serotype distribution

**Figure 2:**
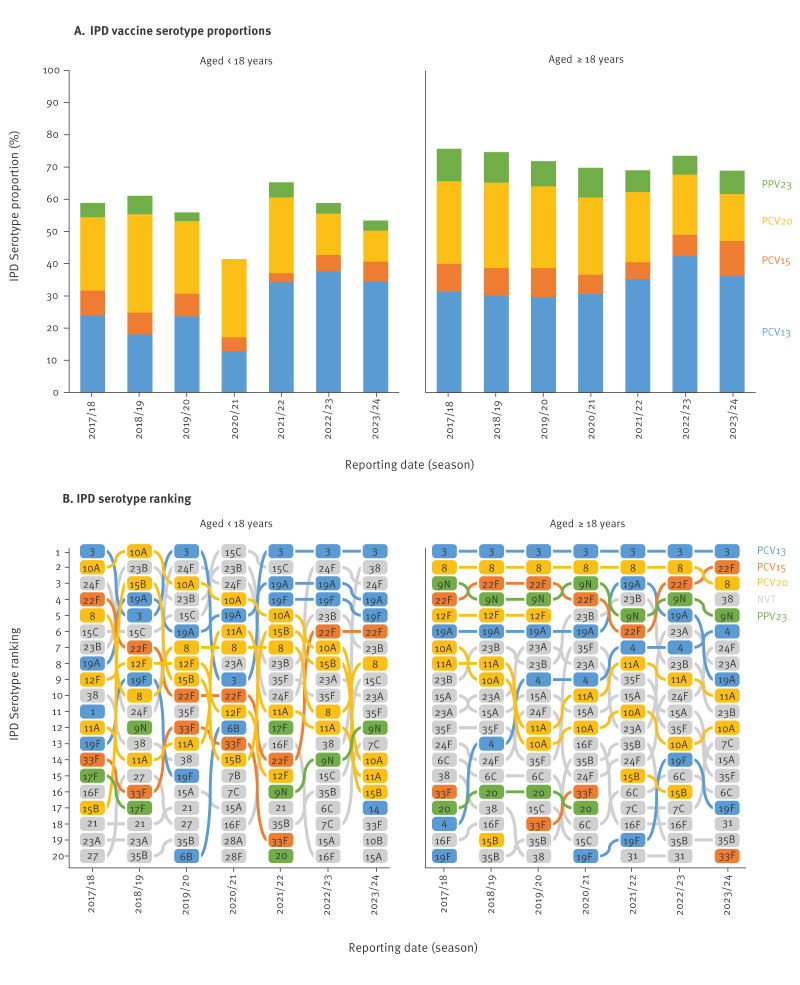
Invasive pneumococcal disease (IPD) serotype distribution, by age group and season, Germany, 2017/18–2023/24

NVT: non-vaccine serotype; PCV: pneumococcal conjugate vaccine; PCV13: serotypes 1, 3, 4, 5, 6A, 6B, 7F, 9V, 14, 18C, 19A, 19F and 23F; PCV15: PCV13 + 22F and 33F; PCV20: PCV15 + 8, 10A, 11A, 12F and 15B; PPV23: 1, 2, 3, 4, 5, 6B, 7F, 8, 9N, 9V, 10A, 11A, 12F, 14, 15B, 17F, 18C, 19A, 19F, 20, 22F, 23F and 33F.

In the pre-pandemic seasons, the proportion of IPD in children and adolescents aged < 18 years, caused by PCV13 serotypes, was on average 21.9% (SD: 2.7) but during the pandemic season, the proportion was 12.9% (9/70 isolates; OR = 0.53; 95% CI: 0.24–1.04). In the post-pandemic seasons, the average proportion increased to 35.4% (SD: 1.4; OR = 3.73; 95% CI: 1.93–8.15) (Figure 2A). The proportion of PCV15 serotypes 22F and 33F in IPD in children and adolescents averaged 7.2% (SD: 0.4) in the pre-pandemic seasons, was 4.3% during the pandemic season (OR = 0.58; 95% CI: 0.14–1.66), 2.8% (OR = 0.38; 95% CI: 0.14–0.84) in 2022/23 and 6.3% (OR = 0.86; 95% CI: 0.50–1.47) in 2023/24. The average proportion of PCV20 serotypes 8, 10A, 11A, 12F and 15B in IPD in children and adolescents was 24.7% (SD: 3.0) between 2017/18 and 2021/22, with a significant decline in the last two seasons (12.8% and 9.7%; OR = 0.39; 95% CI: 0.29–0.51).

Panel A. IPD vaccine serotype proportions for children and adolescents aged < 18 years (left diagram) and adults aged ≥ 18 years (right diagram) coloured by vaccine formulation with PCV13 (blue), PCV15 (orange), PCV20 (yellow) and PPV23 (green).

Panel B. IPD serotype ranking for children and adolescents aged < 18 years (left diagram) and adults aged ≥ 18 years (right diagram) including the 20 most isolated serotypes for each season coloured by vaccine formulation with PCV13 (blue), PCV15 (orange), PCV20 (yellow), PPV23 (green) as well as non-vaccine serotypes (NVTs, grey).

Among adults aged ≥ 18 years, a COVID-19 pandemic-driven reduction in PCV13 IPD was not observed (Figure 2A), with levels stable around an average of 30.4% (SD: 0.7), followed by a significant post-pandemic increase, reaching 42.4% in 2022/23 (OR = 1.68; 95% CI:1.57–1.81). The proportion of adult IPD caused by the two PCV15 serotypes decreased from a pre-pandemic average of 8.7% (SD: 0.2) during the pandemic season (6.0%) and the two subsequent seasons (5.3%; 6.6%; OR = 0.69, 95% CI: 0.61–0.77), followed by a rise above pre-pandemic levels in 2023/24 (10.9%; OR = 1.29; 95% CI: 1.15–1.44). In adults, the five PCV20 serotypes showed a decline from a pre-pandemic average of 25.9% (SD: 0.5) to 14.5% in 2023/24 (OR = 0.49; 95% CI: 0.45–0.53).

#### Serotype ranking

Among children and adolescents aged < 18 years, the IPD serotype ranking fluctuated over the study period, with serotype 3 the most isolated in five of the seven analysed seasons and PCV13 serotypes 19F and 19A among the top five in 3–5 seasons (Figure 2B). Serotype 22F dropped in the rankings after the 2017/18 season but rose to the sixth place in 2022/23 and 2023/24, whereas serotype 33F was not among the top 10 serotypes in children and adolescents during the study period. Regarding serotypes included in PCV20, 10A and 12F dropped in the ranking after 2019/20 and 2020/21, respectively, while 11A, 15B and 8 fluctuated within the top 20, with serotype 8 the dominant PCV20 specific serotype in children and adolescents in 2023/24. Serotypes only included in PPV23 were sporadically seen in children and adolescents and at the lower end of the serotype ranking list with 9N the most common. Of the serotypes not included in any vaccine currently used in Germany, serotypes 24F, 23B and 15C were particularly common among children and adolescents, while serotype 38 increased in 2022/23 and 2023/24.

In adults aged ≥ 18 years, serotype 3 was the dominant PCV13 serotype throughout the entire study period, while 19A remained stable until 2020/21, increased in 2021/22 before dropping again in 2022/23 and 2023/24 (Figure 2B). Serotype 19F ranked at the lower end among adults in the first four seasons and only briefly increased in 2022/23, whereas serotype 4 steadily increased, rising from rank 18 in 2017/18 to rank 6 in 2023/24. Of the two PCV15 serotypes, 22F consistently ranked high, even reaching the second place in 2023/24, whereas 33F appeared in the top 20 of the adult serotype ranking in only four seasons. Of the serotypes exclusively included in PCV20, serotype 8 was the second most frequent serotype associated with IPD in adults in most seasons, being surpassed only in 2023/24 by 22F, while serotypes 11A and 10A were less common, 15B appeared sporadically, and serotype 12F was not among the top 20 after the COVID-19 pandemic season (2019/20). Of the PPV23 serotypes, 9N consistently ranked high in adults, while serotype 20 was found in the lower third of the serotype ranking until 2020/21. Among non-vaccine serotypes, 23A, 23B and 24F were found in higher-ranking positions in adults in several seasons, however, there was also an increase in serotype 38, which moved up to fourth place in 2023/24.

#### Individual serotype dynamics

**Figure 3:**
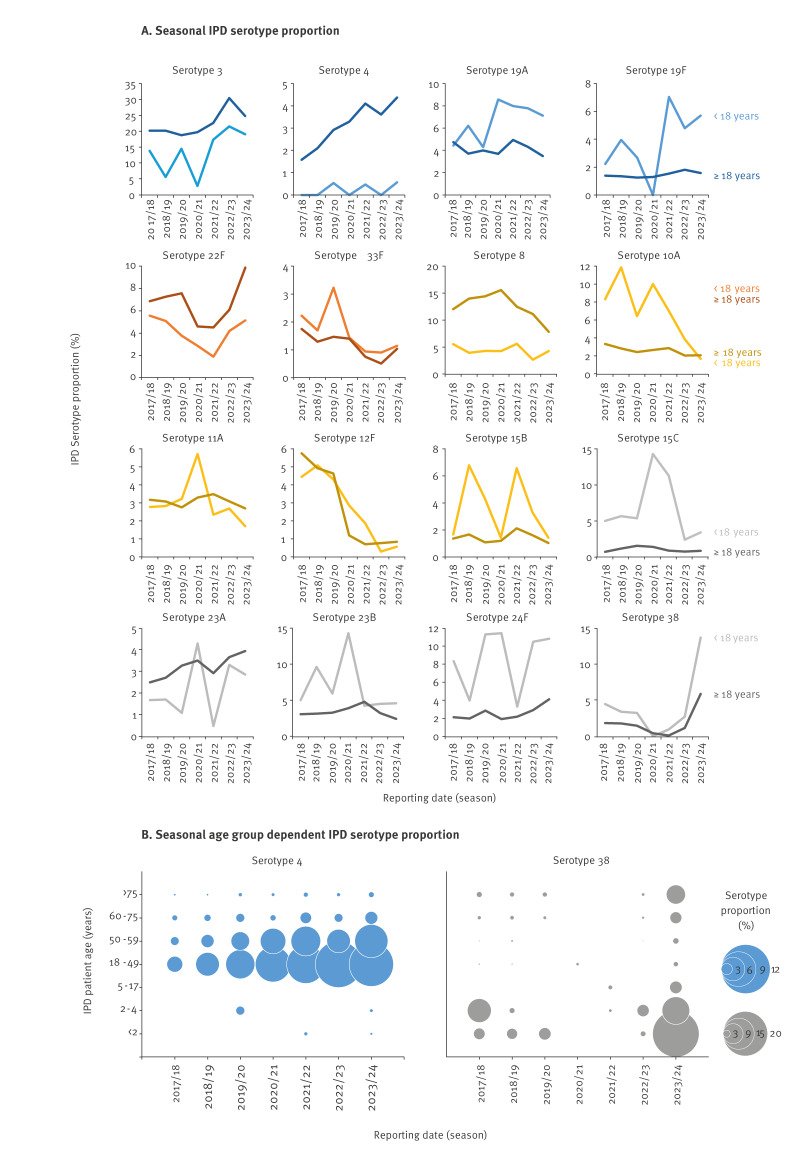
Detailed invasive pneumococcal disease (IPD) serotype dynamics, by season and age group, Germany, 2017/18–2023/24

NVT: non-vaccine serotype; PCV: pneumococcal conjugate vaccine.

Of the serotypes frequently associated with IPD in Germany (Figure 2B), serotype 3 persisted among children and adolescents aged < 18 years over the complete study period with an average proportion of 11.4% (SD: 4.0) in the pre-pandemic seasons, a decrease to 2.9% (OR = 0.23; 95% CI: 0.04–0.75) in the pandemic season (< 2 years: n = 0; 2–4 years: n = 0; 5–17 years: n = 3) and a rise to an average of 19.3% (SD: 1.7; OR = 1.89; 95% CI: 1.39–2.60) in the post-pandemic seasons (Figure 3A). Among adults aged ≥ 18 years, serotype 3 levels averaged 20.3% (SD: 1.3) in the first five seasons of the study period but increased significantly in 2022/23 to 30.5% (OR = 1.71, 95% CI: 1.59–1.85) followed by reduction to 24.8% (OR = 1.29, 95% CI: 1.20–1.39) in 2023/24.

Panel A. Seasonal IPD serotype proportion for children and adolescents aged < 18 years (light coloured lines) and adults aged ≥ 18 years (dark coloured lines) including 16 serotypes, coloured by vaccine formulation in which they are included with PCV13 (blue), PCV15 (orange), PCV20 (yellow) and non-vaccine serotypes (NVT, grey).

Panel B. Seasonal IPD serotype proportion (lend to the right) and seven age groups (left axis) for serotypes 4 and 38 visualised by the diameter of circles, coloured by vaccine formulation in which they are included with PCV13 (blue) and non-vaccine serotypes (NVT, grey).

In addition to serotype 3, PCV13 serotypes 19A and 19F persisted in IPD in children and adolescents aged < 18 years and increased in the post-pandemic seasons (Figure 3A). The average pre-pandemic proportion of 19F was 3.0% (SD: 0.7); it was not detected in the pandemic season and increased in the post-pandemic seasons to an average of 5.8% (SD: 0.9; OR = 1.98; 95% CI: 1.15–3.62). The proportion of serotype 19A averaged 5.0% (SD: 0.9) pre-pandemic in children and adolescents, increased to 8.6% (OR = 1.79, 95% CI: 0.65–4.24) in the pandemic season, and remained around 7.6% (SD: 0.4; OR = 1,57; CI: 1.00–2.52) in the post-pandemic seasons. Among adults aged ≥ 18 years, the proportions of serotypes 19F and 19A have remained at constant levels of 1.5% (SD: 0.2) and 4.1% (SD: 0.5), respectively.

Serotype 4 IPD in children and adolescents aged < 18 years almost disappeared after the introduction of PCV vaccination, with only four cases in the last seven seasons (Figure 3A). However, among adults, the percentage of serotype 4 IPD increased over the study period, from 1.6% (49/3,094) in 2017/18 to 4.4% (250/5,714) in 2023/24 (OR = 2.84; CI: 2.11–3.92), which was most prominent among adults aged 18–49 years (4.2% (15/361)–11.6% (86/742); OR = 3.02; 95% CI: 1.77–5.52) and 50–59 years (2.3% (10/440)–8.7% (60/691); OR = 4.09; 95% CI: 2.17–8.57) (Figure 3B), with a clear focus on male patients (≥ 18 years: 72.6% (530/730) and 18–49 years: 80.6% (216/268).

The PCV15 serotypes 22F and 33F showed some pre- and post-pandemic variations with a proportion of 22F around 5% in children and adolescents and 7% in adults, and 33F between 1% and 2% in both age groups (3.2% in children and adolescents in 2019/20, but only 6/186 cases; Figure 3A). However, a decline of 22F among children and adolescents (2.9%; OR = 0.59; 95% CI: 0.09–2.02) as well as among adults (4.3%; OR = 0.62; 95% CI: 0.45–0.83) was observed in the pandemic season, followed by a rise to 9.9% in adults aged ≥ 18 years in 2023/24 as compared with 7.2% (SD: 0.3) in the pre-pandemic period (OR = 1.42; 95% CI 1.25–1.59).

Of the five serotypes included in PCV20 only, the proportion of serotype 8 decreased among adults aged ≥ 18 years in the post-pandemic seasons (OR = 0.70; 95% CI: 0.64–0.76) (Figure 3A), as did serotype 10A among children and adolescents aged < 18 years (OR = 0.40; 95% CI: 0.26–0.62), whereas the proportion of serotype 11A in children and adolescents was 5.7% (4/70) in the pandemic season (OR: 2.00, CI: 0.56–5.63). Serotype 15B fluctuated between 1.4% and 6.8% over the study period among children and adolescents, while serotype 12F significantly decreased since the pandemic season both among children and adolescents (2017/18 to 2019/20: 4.6%; SD: 0.3; 2021/22 to 2023/24: 0.9%; SD: 0.7; OR = 0.16; 95% CI: 0.07–0.36 and adults: 2017/18 to 2019/20: 5.1%, SD: 0.5; 2021/22 to 2023/24: 0.8%; SD: 0.1; OR = 0.15; 95% CI: 0.12–0.18).

Regarding non-vaccine serotypes (NVT), the IPD proportions of 15C, 23A and 23B among children and adolescents aged < 18 years increased in the pandemic season (14.3%; OR = 2.95; 95% CI: 1.31–6.18), 4.3% (OR = 2.99; 95% CI: 0.64–10.64) and 14.3% (OR = 2.28; 95% CI: 1.03–4.66), respectively, whereas the proportion of all three serotypes remained mostly unchanged among adults aged ≥ 18 years over the study period (Figure 3A).

The proportion of serotype 24F varied among children and adolescents: around 11% in 2019/20, 2020/21, 2022/23 and 2023/24. Whereas in 2017/18, 2018/19 and 2021/22, the proportions were 8.3%, 4,0% and 3.3%, respectively. Among adults, an increase in the proportion of serotype 24F was observed in 2023/24 (4.1%), compared with the previous seasons (2.2%, SD: 0.4; OR = 1.89; 95% CI: 1.56–2.28).

Serotype 38 was rare with only four cases in the pandemic season (Figure 3A). However, in 2023/24, a steep increase was observed among the very young and the very old (Figure 3B). The increase was most prominent in children aged < 2 years, with a proportion of 20.9% (2017/18–2022/23: 3.0%, SD: 2.3; OR = 7.56; 95% CI: 4.13–14.17), in children aged 2–4 years (2017/18–2022/23: 3.4%, SD: 3.8; 2023/24: 12.4%; OR = 3.75; 95% CI: 1.62–8.82) and children and adolescents aged 5–17 years (2017/18–2022/23: 0.3%, SD: 0.8; 2023/24: 5.6%; OR = 17.35; 95% CI: 2.92–329.71). The increase in serotype 38 IPD among adults in 2023/24 compared with the six previous seasons was most prominent in those aged 60–75 years (2017/18–2022/23: 1.1%, SD: 0.7; 2023/24: 5.3%; OR = 4.29; 95% CI: 3.22–5.74) and > 75 years (2017/18–2022/23: 1.5%, SD: 0.9; 2023/24: 8.6%; OR = 5.39; 95% CI: 4.23–6.91) but also significant among 18–49-year-olds (2017/18–2022/23: 0.6%, SD: 0.5; 2023/24: 2.3%; OR = 5.07; 95% CI: 2.35–11.52) and 50–59-year-olds (2017/18–2022/23: 0.4%, SD: 0.4; 2023/24: 2.7%; OR = 5.16; 95% CI: 2.56–10.74).

### Vaccine preventable proportions

#### PCV13, PCV15 and PCV20

**Figure 4:**
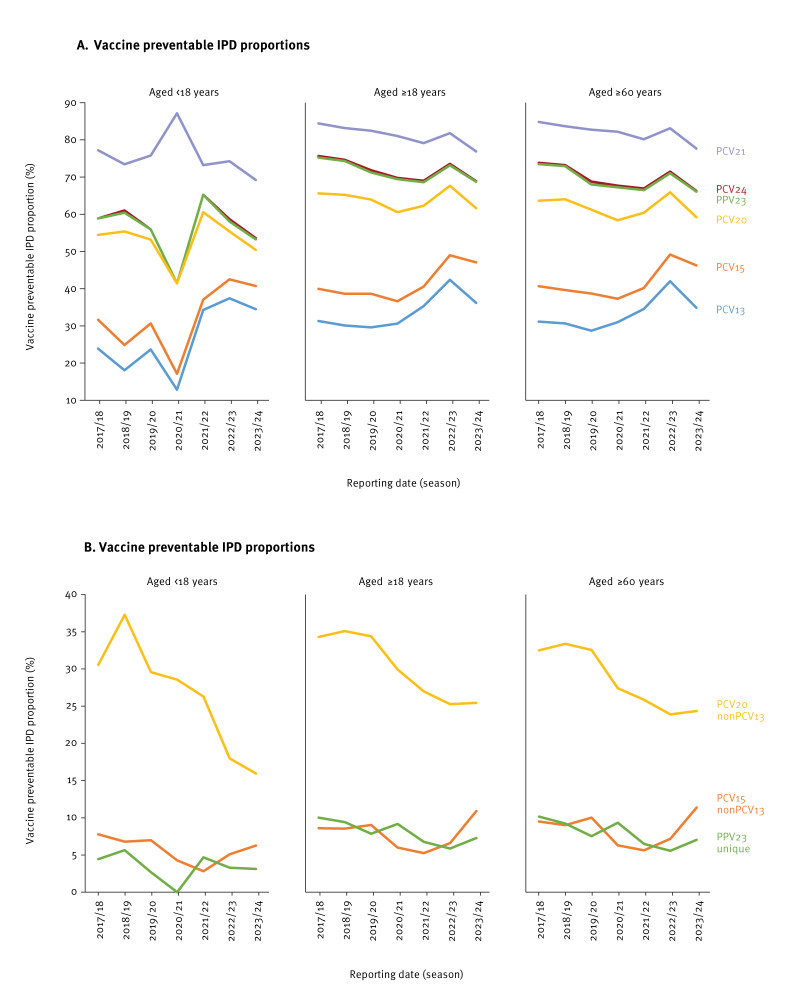
Vaccine preventable invasive pneumococcal disease (IPD), by season and age, Germany, 2017/18–2023/24 (n = 24,122)

NVT: non-vaccine serotype; PCV: pneumococcal conjugate vaccine; PCV13: serotypes 1, 3, 4, 5, 6A, 6B, 7F, 9V, 14, 18C, 19A, 19F and 23F; PCV15: PCV13 + 22F and 33F; PCV20: PCV15 + 8, 10A, 11A, 12F and 15B; PPV23: 1, 2, 3, 4, 5, 6B, 7F, 8, 9N, 9V, 10A, 11A, 12F, 14, 15B, 17F, 18C, 19A, 19F, 20, 22F, 23F and 33F.

Among children and adolescents aged < 18 years, the proportion of cases potentially preventable by PCV13 decreased during the pandemic season to 12.9% and increased in the post-pandemic seasons to an average of 35.4% (SD: 1.4; (Figure 4A), whereas the two additional serotypes in PCV15 were associated with in average 5.7% (SD: 1.6) of IPD cases in the last seven seasons, with lowest rates in 2021/22 (Figure 4), while the seven additional serotypes in PCV20 represented in average 26.6% (SD: 6.9) of IPD cases in the same period, with a decrease in the last two seasons (2022/23: 18.0% (60/334), 2023/24: 16.0% (56/351)).

Panel A. Proportion of IPD potentially preventable by vaccination with PCV13 (blue line), PCV15 (orange line), PCV20 (yellow line), PCV21 (purple line), PCV24 (red line) and PPV23 (green line).

Panel B. Proportion of IPD potentially preventable by vaccination with the two PCV15 nonPCV13 serotypes (orange lines), the seven PCV20 nonPCV13 serotypes (yellow lines) and the four PPV23 unique serotypes (green lines).

Among adults aged ≥ 18 years, PCV13 serotypes also increased in the post-pandemic period, reaching 42.4% (2,102/4,958) in 2022/23 (Figure 4A), while PCV15 additional serotypes were associated with an average of 7.8% (SD: 1.8) of IPD cases over the study period, with the lowest rate in 2021/22 (5.3%, 119/2,266; Figure 4B). The seven additional serotypes in PCV20 were associated with an average of 30.2% (SD: 4.1) of IPD cases in adults, with the lowest values in 2022/23 (25.3%; 1,253/4,958) and 2023/24 (25.4%; 1,454/5,714), again accompanied by an increased proportion of PCV13 serotype IPD.

Among adults eligible for vaccination (≥ 60 years), PCV13 serotypes represented an average of 33.3% (SD: 4.1) of IPD cases over the study period (Figure 4A), while PCV15 serotypes accounted for an average of 41.7% (SD: 4.0) and PCV20 serotypes for an average of 61.8% (SD: 2.6). For the season 2023/24, PCV13, PCV15 and PCV20 vaccination could have prevented 34.9% (1,494/4,281), 46.3% (1,981/4,281) and 59.2% (2,536/4,281) of IPD cases, respectively.

Over the study period, the proportion of IPD associated with the four serotypes unique for PPV23 as compared with PCV20 (2, 9N, 17F, 20) was on average 7.9% (SD: 1.6); Figure 4B).

#### PCV21 and PCV24

In the first six seasons of the study period, PCV21 serotypes accounted for an average of 82.0% (SD: 1.7) of IPD cases among adults aged ≥ 18 years, with a slight decrease to 76.9% (OR = 0.72; 95% CI: 0.67–0.77) in 2023/24. In adults aged ≥ 60 years, these percentages were 82.8% (SD: 1.4) and 77.7% (OR = 0.71; 95% CI: 0.65–0.77) (Figure 4A). A combined strategy of PCV13/PCV15/PCV20 in children and PCV21 in adults, assuming herd protection from children to adults and hypothesising herd protection from adults to children, would have potentially prevented 76.9% of IPD among children and adolescents aged < 18 years and 82.9% of IPD among adults aged ≥ 60 years in 2023/24.

PCV24 would have prevented 53.6% of IPD serotypes among children and adolescents aged < 18 years, 68.9% among adults aged ≥ 18 years and 66.3% among adults aged ≥ 60 years eligible for vaccination, in 2023/24 (Figure 4A).

## Discussion

The analysis of the 24,122 IPD cases reported to the GRLS from July 2017 to June 2024 demonstrates that the majority (68.9%) of IPD cases in a PCV-vaccinating country like Germany occurred in adults aged ≥ 60 years, with only 6.3% of cases reported in children and adolescents. This indicates the success of childhood vaccination but also shows that a reduction in infections among adults could not be achieved, which is in concordance with findings from other European countries [1]. One reason might be the low pneumococcal vaccination rates in adults aged ≥ 60 years in Germany [14].

Over the 7-year study period, a clear reduction in reported IPD case numbers was observed in the COVID-19 pandemic season, which has been reported in many countries worldwide, and was described as consequence of NPIs (face masks, social distancing) interfering with respiratory virus transmission [17,18,28]. The post-pandemic rebound in IPD case numbers was first observed in children [29], whereas among older adults the recovery was delayed, apparently linked to strictly and longer maintained NPIs within this age group. This strong post-pandemic increase above pre-pandemic levels could be the result of enhanced reporting following introduction of obligatory IPD notification in Germany in March 2020, which was delayed and only became effective 2 years later, as corresponding data of the SurvStat@RKI 2.0 system are only available starting from 2022. However, England, which has not introduced any changes in its surveillance system, has reported a comparable post-pandemic IPD increase as have several other countries, like Belgium and Italy [30-32].

Comparing the voluntary GRLS reporting and the mandatory SurvStat notifications for 2022/23 and 2023/24 shows that the GRLS capture rate was remarkably high, especially for children (87%) but also for adults (72%). Including the 10.2% of cases who could not be confirmed as IPD by the GRLS isolate determination, the capture rate for IPD in Germany in 2023/24 as compared with the mandatory notification rates was 98% for children and 80% for adults, suggesting near complete reporting. If all laboratories followed the notification process, which is obligatory by law (Infektionsschutzgesetz), 100% of IPD cases should be notified in SurvStat@RKI 2.0. However, reporting to the GRLS is unlikely to reach 100% of the SurvStat notifications, due to its voluntary nature.

Based on the official notification data, the IPD incidence in Germany was 7.0 per 100,000 among children aged < 5 years, 5.0 per 100,000 among individuals aged 5–64 years and 26.7 per 100,000 among adults aged ≥ 65 years in 2023/24. This is in concordance with pooled incidences of 9.9 per 100,000, 5.3 per 100,000 and 28.5 per 100,000 for the three age groups in 13 European countries in 2018 [1] and a general IPD incidence of 8.13 per 100,000 in England in 2024 [33]. With 6,065 IPD cases analysed from 83.5 million inhabitants (7.3/100,000) in 2023/24, the GRLS surveillance is among the largest worldwide, handling more cases per year than England and Wales (2022/23: 4,598 cases, 60.6 million inhabitants (7.6/100,000) [33], Germany: 5,292 cases, 83.1 million inhabitants (6.2/100,000), the United States (US) (2022: 2,920 cases, 340 million inhabitants, sentinel for 35.3 million inhabitants (0.9/100,000 nationwide, 8.3/100,000 sentinel) [34], Germany: 3,482, 83.1 million inhabitants (4.2/100,000)) or France (2021: 1,382 cases, 67.7 million inhabitants (2.0/100,000) [35], Germany: 1,638 cases, 83.1 million inhabitants (2.0/100,000)).

Increased occurrence of serotype 4 IPD among adults, as observed in Germany in this study, has also been reported from the US [36] and Canada [37], especially among people experiencing homelessness. In Belgium, serotype 4 IPD has increased among younger adults, particularly among males [38], which is in concordance with data from Germany, with a proportion of male patients of 81% among 18–49-year-olds. The almost complete absence of serotype 4 in children and adolescents suggests a circulation among younger adults, not reached by herd protection or a vaccination recommendation. Therefore, a risk group recommendation could be considered, even though people experiencing homelessness are notoriously elusive.

The numbers of serotype 38 IPD cases have been low in Germany in the pre-vaccination and vaccination era, even though this serotype has been described to be associated with high invasive disease potential [39]. The sudden increase of serotype 38 among young children and older adults in 2023/24 is complementary to the age group in which the serotype 4 increase was observed and could be linked to the expansion of already circulating clones in Germany and Poland, but not in the Netherlands [40]. As serotype 38 is not included in any current or envisioned vaccine formulation at any stage of development, the increase in IPD cases associated with this serotype is worrisome and should be carefully monitored. Interestingly, case numbers with serotype 38 remained relatively stable over the first six seasons of our study and were not affected by the COVID-19 pandemic.

Following the introduction of PCV13, the proportion of IPD cases linked to serotype 12F increased [41] and so it was included in PCV20, PCV21 and PCV24. In our study, the proportion of serotype 12F IPD was around 5% across all age groups in the pre-pandemic period. But, since 2020/21, this serotype almost disappeared, with clear impact on the added benefit of PCV20 over PCV13, reducing the amount of IPD cases that could potentially be prevented by this vaccine formulation among children and adolescents from 32.5% in the pre-pandemic period to 16.0% in 2023/24 and from 32.8% to 24.3% among adults aged ≥ 60 years, which is similar to data reported from England [33]. The pandemic-induced reduction of serotype 22F IPD was, in contrast to serotype 12F, associated with a post-pandemic rebound, which was also observed in England [33]

After introduction of childhood vaccination in Germany, only PCV13 serotypes 3, 19F and 19A persisted in IPD among children and adults, which is an indicator of insufficient direct and indirect protection by the current vaccination strategy based on PCV13 and PCV15, probably linked to low uptake, with only three quarters of the children fully vaccinated at the age of 2 years (74%; year of birth 2021) [14]. A large portion of childhood IPD occurred in either not or incompletely vaccinated children [42], showing that the current vaccination rates leave ample room for vaccine serotypes to be carried and transmitted. In the post-pandemic seasons, serotypes 3, 19F and 19A IPD further increased among children, resulting in a statistically significant rise of PCV13 preventable IPD, while among adults, serotypes 3 and 4 were the main drivers of the PCV13 serotype increase, with serotype 3 currently making up one in four cases. Even though childhood vaccination rates in England were much higher (2021/22: 89.3%), a similar post-pandemic expansion of PCV13 serotypes was observed among children and adults, associated with the same serotypes, which was discussed to be linked to the pandemic NPI measures [33].

The additional serotypes in PCV15 and PCV20 were responsible for a considerable amount of IPD in Germany over the study period. The proportion of IPD caused by serotypes 22F and 33F remained rather constant, whereas PCV20-non-PCV15 serotypes decreased in the post-pandemic seasons, in all age groups. This reduction, in adults mainly in serotypes 8 and 12F, in children and adolescents in serotype 12F and to a lesser extent serotype 10A, was accompanied by an increase in PCV13 serotypes, resulting in a slightly reduced proportion of PCV20 preventable IPD. A reduction in serotypes 22F and 33F IPD cases among children aged < 2 years was not observed, probably indicating that it is still too early to detect PCV15 vaccination effects, as it has been specifically recommended since May 2023 only. As the contribution of the four PPV23 unique serotypes (2, 9N, 17F, 20) is modest among adults aged ≥ 60 years, the decision of the German standing committee on vaccination (STIKO) to change the recommendation to PCV20 appears justified. The CAPNETZ cohort supports this view: in 2020, PCV20 serotypes accounted for 64.9% (48/74) of predominantly non-invasive, microbiologically confirmed adult pneumococcal pneumonia, virtually identical to the 61.8% PCV20 preventable IPD in adults ≥ 60 years and underscoring the vaccine’s relevance beyond invasive disease [43].

PCV21 was recently licensed by the European Medicines Agency for adults aged ≥ 18 years and would prevent the highest proportion of serotypes currently associated with adult IPD in Germany. The composition of PCV21 omitted serotypes that have largely been removed from circulation through herd protection by childhood vaccination and replaced them by serotypes more frequently associated with IPD among adults, aiming towards a complementary strategy to suppress a high overall number of serotypes. However, the fact that PCV21 contains neither serotype 4 nor 38, which are currently increasing among adults, could pose a problem to this strategy.

Two new formulations currently under development include 24 serotypes, one based on the multiple antigen-presenting system [44], which represents a new conjugation technique, and another formulation using an enhanced carrier protein [45]. Both formulations include all serotypes of PPV23, plus serotype 6A, consequently a slightly higher proportion of IPD might have been prevented by these vaccines.

Noteworthy limitations of the study include the voluntary base of the GRLS surveillance, which introduces the risk of reporting bias. Due to continuously improving reporting rates over the seven study years, statements on incidence changes for serotypes or serotype groups cannot be made. Nevertheless, data from mandatory laboratory reporting of IPD in Germany introduced in 2020 suggest near complete reporting in the most recent study years, but under-reporting by both systems must be considered. However, incidence levels of IPD in several other European countries are very similar to data presented from Germany.

## Conclusion

National IPD surveillance at the GRLS has reached 87% reporting for children and 72% for adults and in that way, not only provides a solid nationwide representation of case numbers and incidence in Germany but also makes the GRLS one of the largest IPD surveillance systems worldwide. Over the study period from 2017 to 2024, the serotype distribution in IPD varied considerably, mostly due to COVID-19 pandemic related NPIs. PCV13 serotypes gained importance especially among children, which might be related to low vaccination uptake, whereas the overall proportion of PCV20 preventable IPD remained stable at around 50–60%. The rise of serotype 3 and serotype 4 in adult IPD, highlights the importance of direct protection, and supports the current STIKO vaccination strategy for this age group, whereas the sudden appearance of serotype 38 in IPD among young children and older adults illustrates the importance of continuous and comprehensive IPD serotype surveillance.

## Data Availability

All data generated or analysed during this study are included in this article and its Supplement.

## Supplementary Data

135000 Supplementary Material
